# Comprehensive Analysis of Disulfidptosis-Related LncRNAs in Molecular Classification, Immune Microenvironment Characterization and Prognosis of Gastric Cancer

**DOI:** 10.3390/biomedicines11123165

**Published:** 2023-11-28

**Authors:** Kuo Kang, Xuanxuan Li, Yuanhao Peng, Yangying Zhou

**Affiliations:** 1 Department of General Surgery, Xiangya Hospital, Central South University, Changsha 410008, China; kangkuo0825@csu.edu.cn; 2 Hunan Key Laboratory of Precise Diagnosis and Treatment of Gastrointestinal Tumor, Xiangya Hospital, Central South University, Changsha 410008, China; 3 Department of Oncology, Xiangya Hospital, Central South University, Changsha 410008, China; lixx0316@126.com; 4 National Clinical Research Center for Geriatric Disorders, Xiangya Hospital, Central South University, Changsha 410008, China; 5 National Health Council Key Laboratory of Carcinogenesis, Cancer Research Institute and School of Basic Medicine, Central South University, Changsha 410078, China; pengyuanhao1996@163.com

**Keywords:** disulfidptosis, gastric cancer, lncRNAs, risk model, prognosis, immune microenvironment

## Abstract

Background: Disulfidptosis is a novel form of programmed cell death that unveils promising avenues for the exploration of tumor treatment modalities. Gastric cancer (GC) is a malignant tumor characterized by high incidence and mortality rate. However, there has been no systematic study of disulfidptosis-related long noncoding RNAs (DRLs) signature in GC patients. Methods: The lncRNA expression profiles containing 412 GC samples were acquired from the Cancer Genome Atlas (TCGA) database. Differential expression analysis was performed alongside Pearson correlation analysis to identify DRLs. Prognostically significant DRLs were further screened using univariate COX regression analysis. Subsequently, LASSO regression and multifactorial COX regression analyses were employed to establish a risk signature composed of DRLs that exhibit independent prognostic significance. The predictive value of this risk signature was further validated in a test cohort. The ESTIMATE, CIBERSORT and ssGSEA methodologies were utilized to investigate the tumor immune microenvironment of GC populations with different DRLs profiles. Finally, the correlation between DRLs and various GC drug responses was explored. Results: We established a prognostic signature comprising 12 disulfidptosis-related lncRNAs (AC110491.1, AL355574.1, RHPN1-AS1, AOAH-IT1, AP001065.3, MEF2C-AS1, AC016394.2, LINC00705, LINC01952, PART1, TNFRSF10A-AS1, LINC01537). The Kaplan–Meier survival analysis revealed that patients in the high-risk group exhibited a poor prognosis. Both univariate and multivariate COX regression models demonstrated that the DRLs signature was an independent prognostic indicator in GC patients. Furthermore, the signature exhibited accurate predictions of survival at 1-, 3- and 5- years with the area under the curve (AUC) values of 0.708, 0.689 and 0.854, respectively. In addition, we also observed significant associations between the DRLs signature and various clinical variables, distinct immune landscape and drug sensitivity profiles in GC patients. The low-risk group patients may be more likely to benefit from immunotherapy and chemotherapy. Conclusions: Our study investigated the role and potential clinical implications of DRLs in GC. The risk model constructed by DRLs demonstrated high accuracy in predicting the survival outcomes of GC and improving the treatment efficacy for GC patients.

## 1. Introduction

Gastric cancer (GC) is a highly heterogeneous and aggressive malignancy, ranking as the fifth most prevalent cancer in the globe and the third leading cause of cancer-related mortality [1]. Due to the absence of specific early symptoms, the majority of patients are diagnosed at advanced stages, resulting in an unfavorable prognosis [2]. The existing rate of overall survival for patients with early gastric cancer is roughly larger than 60%. In contrast, patients with recurrence or metastasis have a survival rate of only 30 to 50% [3]. 

Patients with GC have made limited therapeutic progress in recent decades, particularly those with multidrug resistance, recurrence or pulmonary metastases [4]. Traditional treatments for gastric cancer include surgery, radiation therapy, chemotherapy and targeted therapy [5]. Currently, the main chemotherapeutic agents for gastric cancer include paclitaxel (paclitaxel and docetaxel), fluorouracil (5-fluorouracil, tegretol, capecitabine), platinum (cisplatin and oxaliplatin), irinotecan and epirubicin. The addition of the anti-HER2 drug trastuzumab to chemotherapy has been shown to improve OS in metastatic HER2-positive patients [6]. Furthermore, immunotherapy is a rapidly growing area of research in gastric cancer, and anti-programmed cell death 1 (PD-1) antibodies, such as nivolumab, sintilimab and camrelizumab, have been demonstrated to improve locally progressed overall survival and progression-free survival in patients with GC [7,8,9]. Therefore, identifying effective predictive biomarkers and a prospective signature is essential for the precision medicine of GC patients.

Disulfidptosis is a novel type of cell death characterized by disulfide stress, which results from the excessive accumulation of intracellular cystine. The excessive accumulation of disulfide also leads to the formation of abnormal disulfide bonds between actin backbone proteins, causing the disintegration of the actin network and subsequent cell death [10]. Thus, disulfidptosis represents a unique mode of cell death distinct from other forms, such as copper-induced cell death and ferroptosis [11]. Liu et al. found that abnormal intracellular disulfide accumulation under glucose starvation conditions can induce a previously uncharacterized form of cell death, distinct from apoptosis and iron atrophy, and this cell death is referred to as disulfidptosis [10]. NcRNA plays an important role in various cellular and physiological functions [12]. Long-chain non-coding RNAs (lncRNAs) refer to ncRNAs that exceed 200 nucleotides in length. These lncRNAs are believed to play crucial regulatory roles, and any disruption in their functioning can result in the development of diseases. Consequently, disulfidptosis presents a potential new therapeutic target for gastric cancer, and the investigation of potential biomarkers related to disulfidptosis is of great clinical importance for patients with gastric cancer.

Among them, an increasing number of studies have demonstrated that long non-coding RNAs (lncRNAs) play a crucial role in the regulation of progressive metastasis and the programmed demise of tumors [13,14]. Numerous lncRNAs have been shown to function as oncogenes, promoting tumor growth and are frequently observed to be overexpressed in cancer cells [15,16,17]. Zhou et al. reported that linc00152 could bind to EGFR and modulate its activity, and that simultaneous suppression of linc00152 and EGFR blockade increased lung cancer patients’ sensitivity to chemotherapeutic agents [18]. Recently, researchers have discovered that the lncRNAs WARS2-AS1 and MKLN1-AS, which are linked to copper death, predict survival and exert an influence on chemotherapy sensitivity in patients with a hepatocellular carcinoma [19]. Currently, it is still unclear whether disulfidptosis-related lncRNAs (DRLs) may act as prognostic candidates for GC and what function they play in GC. Therefore, it is essential to further investigate the significance of DRLs as prognostic signatures for GC patients.

In this study, we applied a comprehensive analysis to construct a prognostic signature consisting of disulfidptosis-related lncRNAs. The DRLs signature was validated as an independent prognostic factor for GC, and a nomogram was developed to guide clinically individualized treatment. Additionally, we investigated the correlation between prognostic features and tumor immune microenvironment and common drug sensitivity of GC to provide valuable insights into the treatment and predictive strategies for GC.

## 2. Methods

### 2.1. Data Collection

Initially, we obtained the clinical data, RNA sequencing raw data and somatic mutation data for 412 GC tumors and 32 normal tissue samples from the TCGA online database via R package “TCGAbiolinks” (https://portal.gdc.cancer.gov/repository, accessed on 15 April 2023). The mRNA data were then transformed into TPM format and log2 transformed to standardize them. For the sample enrollment, we complied with the following criteria: (1) a pathological diagnosis of gastric adenocarcinoma (adenocarcinomas, cystic, mucinous and serous neoplasms); and (2) full clinical and sequencing data (age, sex, grade, survival status, etc.). Patients with a follow-up period of fewer than 30 days were also not included. Supplementary Table S1 summarizes the clinicopathologic characteristics (including age, sex, stage and grading) of patients in the TCGA database. Next, RNA-seq data were extracted from the standard FPKM format using the Perl programming language (Strawberry-perl-version 5.30.0; https://www.perl.org, accessed on 15 April 2023). In addition, Perl was used to extract pathological information, age, staging and survival data from clinical data.

### 2.2. Identification of Disulfidptosis-Related lncRNAs and Differential Expression Analysis in GC

Through previous published studies, we identified 78 disulfidptosis-related genes (Supplementary Table S2) [10]. To obtain the disulfidptosis-related gene expression matrix, we utilized the R packages “BiocManager” and “limma”. Then Pearson co-expression analysis was performed based on the expression of disulfidptosis-related genes and lncRNAs (|Pearson R| > 0.4 and *p*-value < 0.001) [19]. To compare the expression levels of DRLs between GC tumor and normal tissues, we employed the Wilcoxon test. We then performed differential expression analysis using the “limma” package, with a screening threshold set to |log2 FC| ≥ 1 and FDR < 0.05 [20]. The heatmap of differentially expressed lncRNAs was created using the “pheatmap”.

### 2.3. The Construction of the DRLs Signature

After screening the differential lncRNAs, GC patients were randomly assigned to the training set and testing set using the “caret” package in a ratio of 1 to 1. The training set was utilized to construct the DRL’s predictive signature. Validation of the model was based on testing and complete collections. 

Next, we used the “survival” package to identify DRLs associated with GC patients’ prognosis by univariate COX analysis (*p* < 0.05). To filter out the best potential DRLs prognostic signature among the DRLs, we employed the least absolute shrinkage and selection operator (LASSO) regression and multi-variable COX regression analysis. The resulting models were visualized using the packages “survival”, “survminer”, “glmnet” and “timeROC”. Finally, the disulfidptosis-related prognostic risk score for each GC patient was calculated according to the following formula: risk score = ∑ (Coefi × Expi). Here, Coefi and Expi represent the corresponding coefficients and expression levels of each lncRNA, respectively. 

Based on the median risk score, the patients in the training set were divided into two subgroups: the low-risk group and the high-risk group. The Kaplan–Meier (K–M) survival analysis was performed to compare the overall survival (OS) and progression-free survival (PFS) between these two distinct risk groups. Hazard ratios (HRs) and log-rank *p*-values, along with their corresponding 95% confidence intervals (CI), were calculated to assess the significance of the differences in survival outcomes between the groups [21].

To further validate the prognostic value of the constructed risk model, we employed the Chi-square test to analyze the relationship between the model and various clinicopathological features. Additionally, receiver operating characteristic (ROC) curves, along with the calculation of the C-index, were utilized to measure the accuracy of the DRLs prognostic signature. The packages “survminer,” “survival,” “timeROC”, “pec” and “rms,” were used to perform these analyses and generate the relevant visualization [22].

### 2.4. Prognostic and Independent Analysis

To exclude potential confounding effects of other clinicopathological information, we performed subgroup analyses of survival via the clinical data, including age, gender and stage. Subsequently, univariate and multivariate COX analyses were employed to determine if the new DRLs signatures possessed independent predictive potential.

### 2.5. Risk Score Signature-Based Establishment and Evaluation of Nomogram in GC 

Using risk score characteristics and clinicopathological parameters, nomograms predicting 1-, 3- and 5-year survival of GC patients were developed by the “survcomp”, “survival”, “regplot” and “rms” packages [23]. Calibration curves were employed to evaluate the consistency between the expected survival rate predicted by the prognostic model and the observed survival rate. 

### 2.6. Functional Enrichment Analysis 

To further investigate the functional enrichment of genes in distinct risk groups, we first identified differentially expressed genes between the two different risk subgroups (|log2 FC| > 1, FDR < 0.05). If Log2 FC (fold change) is greater than 1 or less than −1 (|log2 FC| > 1), we think that the gene is expressed significantly different in two risk groups. Furthermore, the false discovery rate (FDR) should be less than 0.05 [24,25]. Then, based on the Gene Ontology (GO) enrichment analysis, the Cellular Components (CC), Molecular Functions (MF) and Biological Processes (BP) between high-risk and low-risk groups were performed by the “clusterProfiler” package. Additionally, Gene Set Enrichment Analysis (GSEA) and the Kyoto Encyclopedia of Genes and Genomes (KEGG) pathway analysis were utilized to determine the characteristic signaling pathways in various risk groups. The significance levels were determined based on the adjusted *p*-value being less than 0.05 [26].

### 2.7. Assessment of Tumor Microenvironment (TME) and Immune Cell Infiltration Levels 

To compare the proportions of tumor infiltration immune cells between the high-risk and low-risk groups, the CIBERSORT algorithm, which containing 22 different types of immune cells (including B cells naïve, B cells memory, Plasma cells, T cell CD8, Tcell CD4 naïve, T cells CD4 memory resting, T cells CD4 memory activated, T cells foclicular helper, T cells regulatory, T cells gamma delta, NK cells resting, NK cells activated, Monocytes, Macrophages, Macrophages M0, Macrophages M1, Macrophages M2, Dendritic cells resting, Dendritic cells activated, Mast cells resting, Mast cells activated, Eosinphils and Neutrophils), was used to calculate the infiltration scores. By applying the ESTIMATE algorithm, the StromalScore, ImmuneScore and ESTIMATEScore were evaluated between high-risk and low-risk groups. To investigate the immune function and cell subgroups, we utilized Single-sample Gene Set Enrichment Analysis (ssGSEA) [27].

### 2.8. Tumor Somatic Mutation and Tumor Mutation Burden Analysis

TCGA mutation data were downloaded and analyzed via the MAFTOOLS application to determine differences in somatic mutation frequencies between the DRLs risk groups [28]. We have selected the genes with the highest mutation frequencies to present in the form of a waterfall chart. In the meantime, we continue to investigate the association between survival and risk scores and tumor mutation burden (TMB). Furthermore, we combined TMB status and risk signatures to operate stratified analyses. 

### 2.9. The Effect of DRLs on Immunotherapy for GC

The Biomarker Exploration for Solid Tumors (BEST) database enabled us to explore stable biomarkers in multiple separate queues. We further used the BEST database to further explore the effect of disulfide death-related lncRNAs on immunotherapy for gastric cancer. 

### 2.10. The Potential Drug Sensitivity Prediction in GC

To find out if the risk model provides any guiding values for the clinical decision-making process, we used the “pRophetic” software package (version 3.3) to calculate the IC50 of commonly used anti-cancer drugs, with lower IC50 indicating higher sensitivity to the drug. Sensitivity to drugs in distinct risk groups was compared using limma and ggpubr. By screening for potential therapeutic drugs, we examined the CMap database using the differentially expressed genes according to the disulfidptosis-associated risk signature. We obtained a correlation score (−100,100) according to the enrichment analysis of differentially expressed genes. In this study, we utilized small molecular compounds with a correlation score of <−80 as the analytical threshold [29].

### 2.11. Statistical Analysis 

The data statistics and graphical graphing were carried out utilizing the R software (version 4.1.2) and Perl programming language (Strawberry-perl-version 5.30.0; https://www.perl.org, accessed on 16 April 2023). Using Kaplan–Meier curves, survival rates between groups were compared. Using univariate and multivariate Cox regression, independent factors influencing prognosis were analyzed. The *p* < 0.05 was considered statistically significant. 

## 3. Results

### 3.1. Identification of Disulfidptosis-Related Differentially Expressed lncRNAs in Gastric Cancer and Construction of Risk Model

**Figure 1:**
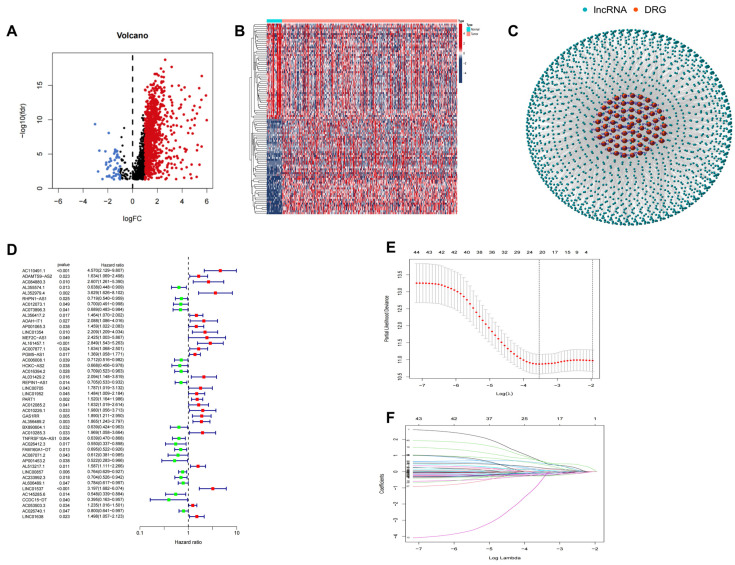
Identification of disulfidptosis-related lncRNAs in gastric cancer and construction of a prognostic signature. (**A**,**B**) Volcano plot and heatmap of differentially expressed disulfidptosis-related lncRNAs in GC. (**C**) Coexpression analysis of lncRNAs and disulfidptosis-related genes. (**D**) Disulfidptosis-related prognostic lncRNAs were obtained via univariate COX regression analysis. (**E**,**F**) Disulfidptosis−related prognostic signature was identified via the LASSO COX regression analysis.

The research flow is shown in Supplementary Figure S1. Firstly, RNA-seq data from GC patients were downloaded from the TCGA database, and the mRNA and lncRNA expression data were distinguished by correction and normalization processes. We obtained 78 disulfide-related genes and screened the differentially expressed lncRNAs between tumor and normal samples by setting a threshold of |Log2 FC| > 1, *p* < 0.05, followed by a correlation coefficient >0.4, *p* < 0.001 screening to finally obtain 999 disulfide-related lncRNAs (Figure 1A,B, Supplementary Tables S3 and S4). Figure 1C showed the coexpression network of disulfidptosis-related genes and disulfidptosis-related lncRNAs.

Clinical and survival data of GC patients were obtained from the TCGA database, and patients with OS deletion were excluded. We identified 22 lncRNAs associated with the prognosis in GC patients via univariate Cox regression analysis among the screened bi-sulfide death-associated lncRNAs (Figure 1D). Subsequently, we screened out 12 critical genes by employing LASSO and multifactorial COX analyses on prognosis-related genes (Figure 1E,F). The risk score for each GC patient was calculated using the following formula: Risk score = (2.120289225 × AC110491.1) + (−0.394086164 × AL355574.1) + (−0.373467544 × RHPN1-AS1) + (1.245072128 × AOAH-IT1) + (0.450022303 × AP001065.3) + (−3.793874086 × MEF2C-AS1) + (−0.520167444 × AC016394.2) +(0.764878872 × LINC00705) + (0.7858314 × LINC01952) + (0.382918529 × PART1) + (−0.408911862 × TNFRSF10A-AS1) + (1.78093689 × LINC01537).

### 3.2. Prognostic Analysis and Internal Validation of DRLs Signature

**Figure 2:**
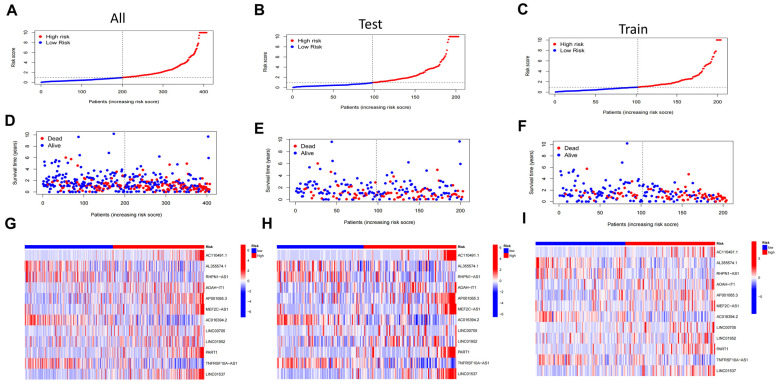
Construction of disulfidptosis-related lncRNAs (DRLs) signature. (**A**–**C**) Distribution of risk scores in entire set, training set and testing set. (**D**–**F**) survival status. (**G**–**I**) The heatmap showed the expression trend of the different risk score in the entire set, training set and testing set.

Based on the algorithm, the risk score for each patient was determined, and the patients were divided into low-risk and high-risk groups using the median risk score, and the survival differences between the two groups were compared in the training set, test set and ensemble, respectively. We observed that the risk distribution scores and the patients’ survival status plots showed a distinct pattern. The death cases were predominantly distributed in the high-risk group, indicating that patients with higher risk scores had a higher likelihood of experiencing poor survival outcomes. Conversely, the survival cases were primarily concentrated in the low-risk group (Figure 2A–F). The heatmap depicted the differential expression levels of 12 DRLs in the three cohorts (Figure 2G–I). 

**Figure 3:**
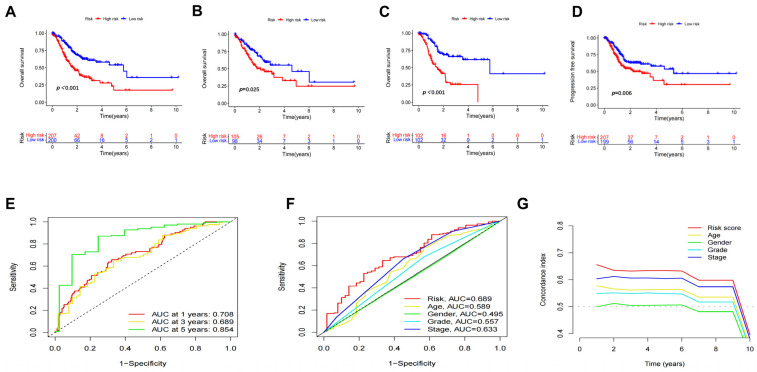
Prognostic analysis of DRLs for gastric cancer patients. (**A**–**C**) Kaplan–Meier survival analysis of overall survival of risk model in entire set, training set and testing set. (**D**) Kaplan–Meier survival analysis of PFS. (**E**) The 1-, 2- and 3- year ROC curves. (**F**) ROC curves comparing predictive risk models with clinicopathological characteristics. (**G**) C-index ROC curve of the risk model.

In addition, K–M survival analysis showed that patients classified as low-risk had a significantly better OS compared to those in the high-risk group (Figure 3A–C). The PFS analysis revealed that the high-risk group exhibited a poorer PFS compared to the low-risk group (Figure 3D). The ROC curves demonstrated the diagnostic validity of our established DRLs signature for 1, 3 and 5 years OS with AUC values of 0.708, 0.689 and 0.854, respectively (Figure 3E). Additionally, the predictive value of the risk score (AUC = 0.689) was significantly higher than other clinicopathological variables when compared with other clinical factors, such as age (AUC = 0.589), gender (AUC = 0.495), grade (AUC = 0.557), stage (AUC = 0.633) (Figure 3F). The C-index curve also revealed that the DRLs signature exhibited a better consistency compared with other factors (Figure 3G).

### 3.3. DRLs Signature as an Independent Risk Factor in GC

**Figure 4:**
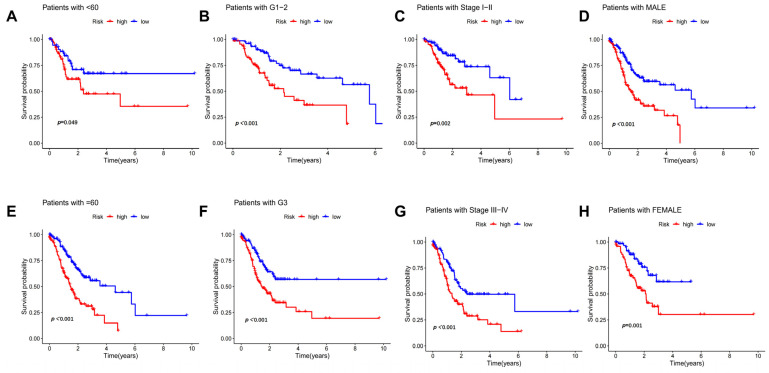
Correlation analysis between the DRLs signature and different clinicopathological characteristics. Kaplan–Meier survival curves for high-risk and low-risk patients in subgroups of different age (**A**,**E**), grade (**B**,**F**), stage (**C**,**G**) and gender (**D**,**H**).

To examine whether clinicopathological characteristics had an impact on the predicted outcome of prognosis, we compared the prognostic value of the risk model in different clinicopathological subgroups of GC (age, gender and T-stage). As illustrated in Figure 4A–H, our results revealed that OS was worse in the high-risk patients than in the low-risk patients in all of the different clinical variable subgroups.

**Figure 5:**
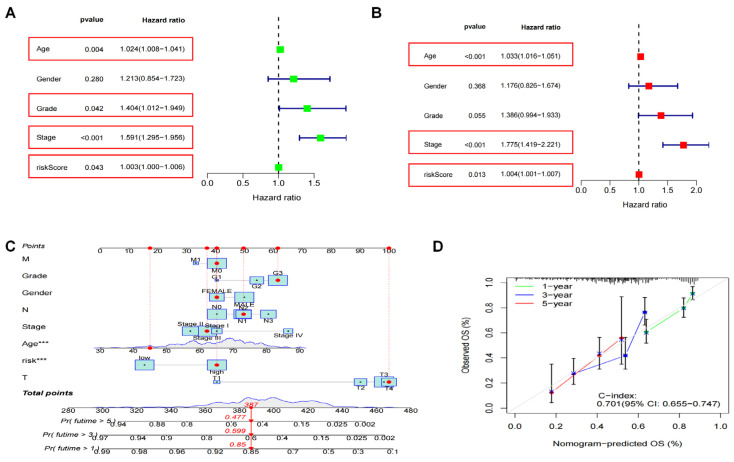
Independent prognostic analysis and prognostic nomogram establishment. (**A**) The forest plots for univariate COX regression analysis of clinical feature and risk score with OS. (**B**) Multivariate COX regression analysis. (**C**) A nomogram to predict GC patients’ outcomes in 1, 3 and 5 years. (*** *p* < 0.001) (**D**) The calibration curves. The red box means significant clinicopathological features with *p* < 0.05.

Next, we compared the prognostic value of the risk model across distinct clinicopathological subgroups (age, gender and T-stage) of GC. As displayed in Figure 5A, univariate COX regression analysis revealed OS in GC patients was substantially associated with age, grade, stage and risk score Age (HR:1.033, 95%CI 1.016–1.051), stage (HR:1.775, 95%CI 1.419–2.221) and risk score (HR:1.004, 95%CI 1.001–1.007) were identified as independent risk factors influencing the prognosis of GC patients via multifactorial COX regression analysis (Figure 5B). Our results presented that the high-risk group had unfavorable OS compared to the low-risk group across all different clinical variable subgroups. 

### 3.4. The Construction and Validation of the Nomogram

To apply the risk model to the clinical setting, we established a disulfidptosis-related prognostic line graph model to predict OS at 1-, 3- and 5-years based on clinical characteristics including age, sex and DRLs risk score (Figure 5C). The calibration curves revealed good consistency between our nomogram and the observed survival probabilities at 1-, 3- and 5-years (Figure 5D). Therefore, these findings indicated that the DRL’s prognostic signature was reliable and stable, and that it might be used to guide clinical treatment of GC patients.

### 3.5. Functional Enrichment Analysis of the DRLs Signature 

**Figure 6:**
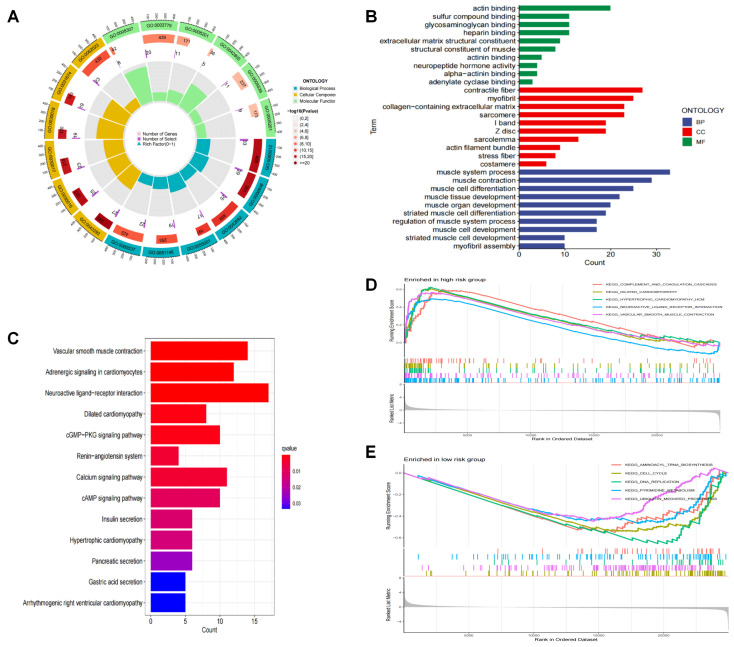
Functional analysis in the different risk groups. (**A**,**B**) GO enrichment analysis of different risk groups. (**C**) KEGG enrichment analysis of different risk groups (**D**,**E**) Gene set enrichment analysis (GSEA) in the high-risk group and low-risk group.

To investigate the underlying biological functions associated with differential genes between high- and low-risk groups, we performed GO and KEGG pathway analyses using the limma package (*p* adjust < 0.05, |log2 (fold change)| ≥ 1). After that, we utilized the “clusterprofiler” R package to perform GO and KEGG enrichment analysis so that we could investigate the DEGs of biological features. As demonstrated by BP terminology, DEGs contributed largely to the muscle system. In the field of CC, contractile fiber, collagen-containing extracellular matrix and myofibril were significantly enriched. The enrichment for MFs was related to actin binding, sulfur compound binding and glycosaminoglycan binding (Figure 6A,B). Furthermore, KEGG analysis revealed that DEGs of high- and low-risk groups were enriched in vascular smooth muscle contraction, neuroactive ligand-receptor interaction and adrenergic signaling in cardiomyocytes (Figure 6C). In addition, the GSEA results revealed that hypertrophic or dilated cardiomyopathy was activated in the high-risk group, while DNA replication and aminoacyl tRNA biosynthesis were significantly enriched in the low-risk group (Figure 6D,E).

### 3.6. Gene Mutation Landscape 

**Figure 7:**
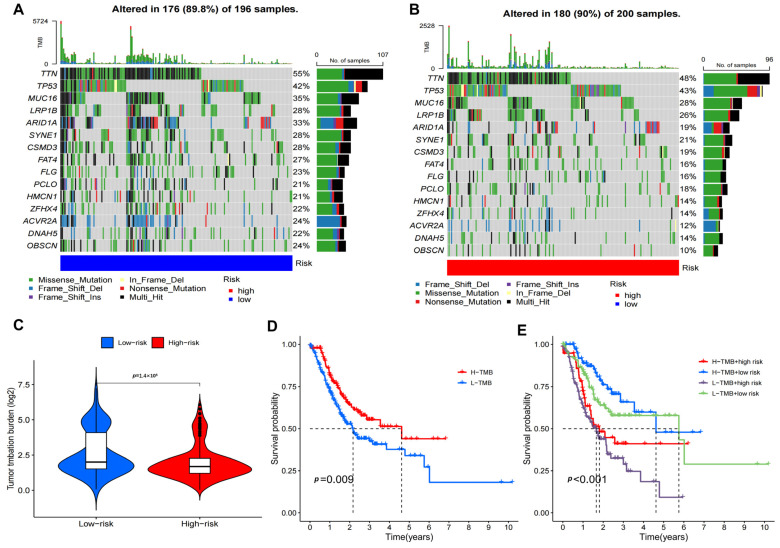
Mutation landscapes of DRLs signature. (**A**,**B**) Waterfall chart showing the mutants status in the high DRLs and low DRLs score group. (**C**) TMB status in high risk and low risk groups. (**D**) The K–M curves show survival status and survival time in high-TMB and low-TMB groups. (**E**) Stratified survival analysis.

To investigate the mutation status differences between the high-risk and low-risk groups, we compared somatic mutations observed in the two groups and identified the top 15 genes with the highest mutation frequency. The results showed that the mutation frequency in both groups was almost 90% (Figure 7A,B). *TTN*, *TP53*, *MUC16*, *LRP1B* and *ARID1A* are the top five genes with relatively high mutation rates in the two groups. Oncogenes such as MUC16 displayed a comparatively lower mutation rate in the high-risk group (24% vs. 29%), in contrast to tumor suppressor genes such as *TP53*, which had a relatively higher mutation rate in the high-risk group (65% vs. 55%). In the high-risk population, mutations were more prevalent in *TTN*, *FAT1* and *SETD2*. In addition, we analyzed the correlation between risk scores and TMBs. We discovered that the low-risk group showed a higher mutation burden compared to the high-risk group (Figure 7C). Survival analyses demonstrated that the patients with a high TMB had favorable OS (Figure 7D). In addition, stratified survival analyses indicated that in the high-TMB subgroup, patients in the low-risk group have better survival outcomes compared with those in the high-risk group (Figure 7E).

### 3.7. Tumor Immune Microenvironment Landscape in High- and Low-Risk Groups

**Figure 8:**
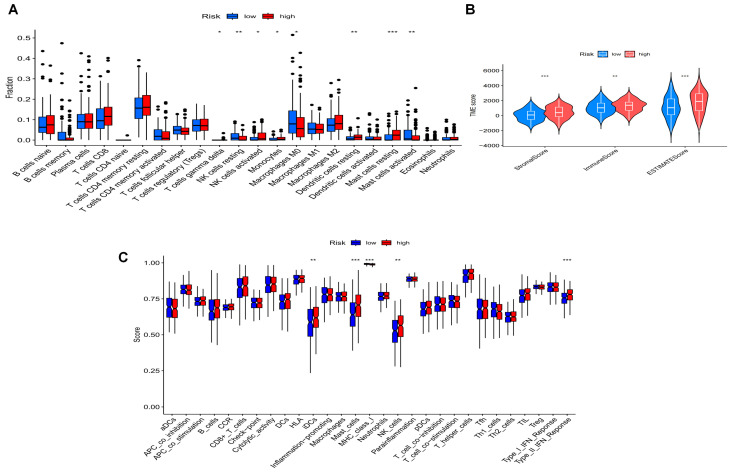
Immune landscape of the DRLs signature. (**A**) Correlation between DRLs signature and tumor immune infiltrating cells. (**B**) Tumor microenvironment analysis between the high- and low-risk groups. (**C**) The relationship between the high- and low-risk groups in the scores of immune cell ratios and immune-related functions. * *p* < 0.05, ** *p* < 0.01, and *** *p* < 0.001.

The tumor immune microenvironment plays a vital role in influencing the effectiveness of immunotherapy. To further investigate the association between disulfidptosis-related signatures and antitumor immunity in GC patients, we analyzed the relative proportion of immune cell infiltration of all GC patients by employing the CIBERSORT algorithm. Figure 8A depicts the proportion of each typical immune cell, and the results demonstrated that the differences between high- and low-risk groups were concentrated in gamma delta T cells, activated NK cells, resting NK cells, M0 macrophages, monocytes, resting mast cells, resting dendritic cells and activated mast cells. Patients in the high-risk group had a higher proportion of activated NK cells, monocytes and resting dendritic cells. 

To determine the differences in infiltrating immune cells between the two groups, we compared the StromalScore (stromal cells in tumor tissue), the ImmuneScore (immune cell infiltration in tumor tissue) and the ESTIMATEScore (stromal and sum of immune scores). We explored that the low-risk group of GC patients had higher TME scores (estimate score, immune score and stromal score) compared to the high-risk group (*p* < 0.001) (Figure 8B). The ssGSEA analysis also showed significant differences between the low- and high-risk groups in the immune-related functions and scores of immune cell ratios (Figure 8C).

### 3.8. The Effect of DRLs on Immunotherapy for GC

Among the signature DRLs, we found that MEF2C-AS1 was highly expressed in people who did not respond to anti-CTLA-4 in Nathanson cohort (Supplementary Figure S2A). In the Wolf cohort, the expressions of PART1 (*p* = 0.0032) and LINC00705 (*p* = 0.024) in anti-PD-1/PD-L1 responders were both increased (Supplementary Figure S2B,C). In the Riaz cohort, the expression of RHPN1-AS1 in anti-PD-1/CTLA-4 non-responders was elevated (*p* = 0.00062, Supplementary Figure S2D). Furthermore, the ROC curve of AUC values of MEF2C-AS1, PART1, LINC00705 and RHPN1-AS1 were 0.719, 0.678, 0.637, and 0.734, respectively (Supplementary Figure S2E–H). These results suggested that DRLs had a good performance in distinguishing anti-PD-1/PD-L1 responders and non-responders in gastric cancer.

### 3.9. Drug Sensitivity Analysis in DRLs Risk Model 

**Figure 9:**
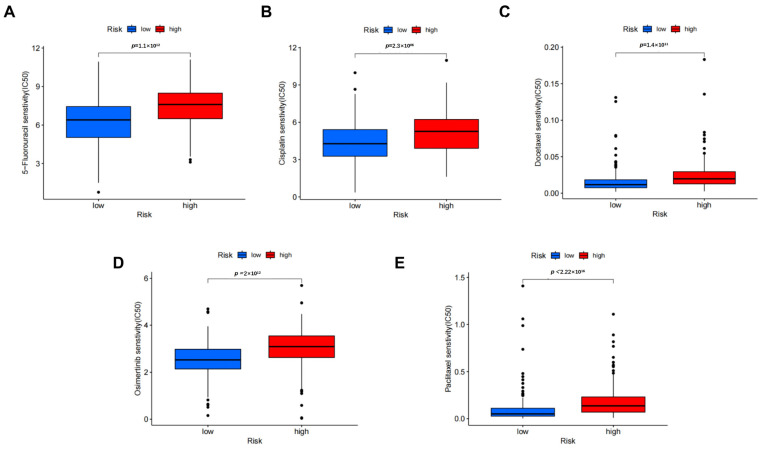
Drug sensitivity analysis in DRLs signature in GC. The great differences in drug sensitivity between high- and low-risk groups for five drugs: 5-Fluorouracil (**A**), Cisplatin (**B**), Docetaxel (**C**), Oxaliplatin (**D**) and Paclitaxel (**E**).

To assess the association of DRLs risk signature and drug sensitivity, we utilized the prophetic package to determine the IC50 of drugs in both groups. We screened gastric cancer-related drugs, including 5-Fluorouracil, Cisplatin, Oxaliplatin, Docetaxel and Paclitaxel g. The IC50 for 5-Fluorouracil (Figure 9A), Cisplatin (Figure 9B), Docetaxel (Figure 9C), Oxaliplatin (Figure 9D) and Paclitaxel (Figure 9E) was elevated with increasing risk scores, which suggested that GC patients in the low-risk group demonstrated a higher likelihood of benefiting from these chemotherapeutic drugs. Therefore, the DRLs risk model may help guide individualized treatment for GC patients.

We further screened potential small molecular compounds for the treatment of gastric cancer from the CMap database. These potential small molecule drugs were ranked and screened based on their relevance scores (Supplementary Table S5). The results showed some potential small molecule drugs for the treatment of gastric cancer, such as lapatinib, Brivanib, tyrphostin-B44, etc.

## 4. Discussion

Due to its high mortality and recurrence rates, gastric cancer is extremely harmful to people’s health [30]. Despite the current improvements in gastric cancer treatment and diagnosis, and the application of new drugs that have dramatically changed the treatment paradigm, the proportion of individuals who can benefit from it remains limited. Therefore, we need to identify reliable biomarkers and develop a prognostic model to better stratify and customize patients with GC.

LncRNAs play crucial roles in diverse pathophysiological processes, and are capable of regulating epigenetic regulation, transcriptional and post-transcriptional gene expression, which are closely related to tumor malignant invasion, proliferation and metastasis [31,32]. Dai et al. showed that LncRNA UCA1 can activate PI3K/AKT pathway through EZH2, enhance cisplatin resistance and regulate apoptosis in GC [33]. It was also revealed that Lnc-ASNR can have oncogenic effects by regulating the miR-519E-5p/FGFR2 pathway in GC [34]. Recently, a new cell death mode known as disulfidptosis has been proposed, and it has been shown that massive disulfide bond accumulation leads to aberrant disulfide bond cross-linking between actin cytoskeletal proteins and cytoskeletal contraction, which induces cell death. This new form of cell death cannot be inhibited by drugs typically used to inhibit cell death, nor can it be prevented by knocking down ferroptosis/apoptosis key genes. A deeper understanding of disulfidptosis will provide new insights into fundamental cellular homeostasis and facilitate the development of innovative therapies for disease treatment. There have been some studies reporting on the role of disulfidptosis-associated long non-coding RNAs (lncRNAs) in breast cancer, cervical carcinoma and colon cancer [35,36,37]. Xue et al. found that the lncRNA model associated with disulfidptosis was able to independently predict the prognosis of COAD patients [37]. Liu et al. constructed a disulfidptosis-related lncRNAs prognostic model in cervical cancer and demonstrated that this prognostic signature had good predictive performance and provided biomarkers for personalized treatment of cervical cancer [35]. Similar to previous studies, our study incorporated disulfidptosis-associated lncRNAs into the signature construction, and demonstrated good performance in predicting the survival outcomes and treatment efficacy of GC. To the best of our knowledge, it was the first study in identifying the prognostic signature of disulfidptosis-associated lncRNAs in gastric cancer, and the new DRLs model provided new insights for understanding the development and progression of GC and guiding individualized treatment strategies for GC patients. 

First, we comprehensively explored the DRLs expression in GC, and revealed that DRLs were differentially expressed in tumor tissues versus normal tissues. A total of 999 lncRNAs were identified as DRLs by Pearson correlation and differential expression analysis. Among these correlated lncRNAs, 22 lncRNAs with prognostic relevance to GC were acquired via univariate COX analysis. Then, LASSO regression and multifactorial COX regression analysis were applied to screen out 12 significant lncRNAs and established a DRLs prognostic signature. The predictive value of this new signature was further assessed via survival analysis, ROC curves and internal validation. Previous studies have reported that AL355574.1 and TNFRSF10A-AS1 were identified as glycolysis-associated lncRNAs associated with poor prognosis of gastric cancer and developed as prognostic markers for the prediction of gastric cancer [38]. In addition, Luo et al.’s study revealed that suppression of MEF2C-AS1 increased the invasiveness of gastric cancer [39]. These findings were in line with our explorations. However, no study has been reported on the effects of AC110491.1, AOAH-IT1, AP001065.3, LINC00705 and LINC01952 on cancer prognosis, and need to be further investigated.

We also discovered that this risk model performed well in predicting the prognosis of GC patients by building a nomogram. The clinicopathological features, including age, stage, sex and risk score, were identified as independent risk factors for patients’ OS. In this study, we presented a novel DRLs signature that could be used to identify promising prognostic indicators and facilitate the development of an individualized therapeutic strategy. In addition, we performed gene enrichment analysis of the differential genes in the high- and low-risk groups and found that the biological functions in which the differential genes were involved were related to sulfur compound binding, which is consistent with previous findings [10].The effect of disulfidptosis on the immune properties of tumors remains unclear. Yang et al. [40] constructed a prognostic model based on disulfidptosis-associated genes in hepatocellular carcinoma and revealed that the signature was correlated with immunotherapeutic target genes (SLC7A11 and SLC3A2). It has been reported that IL-1 β increased the level of SLC7A11, then up-regulated the expression of PD-L1 and CSF1 by regulating α-KG/HIF1 α and promoted the infiltration of tumor-related macrophages and myeloid suppressor cells to increase the metastasis of hepatocellular carcinoma [41]. In addition, the release of interferon-γ from activated CD8^+^ T cells can inhibit SLC7A11, thus further activating the ferroptosis pathway to play an anti-tumor effect. Therefore, the inhibition of SLC7A11 can enhance the anti-tumor effect of immune cells [42]. In addition, we further analyzed the correlation between *SLC7A11* and the risk model established in this study and found that were negatively correlated (*R* = −0.29, *p* = 1.8 × 10^−8^, Supplementary Figure S3), suggesting that *SLC7A11* may also play a certain role in the process of disulfidptosis, and it still needs to be further investigated. To further assess the tumor immune microenvironment in GC, we analyzed the differences in tumor-infiltrating immune cells and tumor microenvironment scores among distinct DRLs risk subgroups. A previous study has demonstrated that patients with higher stromal and immune scores had a favorable prognosis. Patients classified under the low-risk subtype exhibited higher levels of Stroma score, Immune score and ESTIMATE score compared to patients belonging to the high-risk group. According to the findings of CIBERSORT, the infiltration of the majority of immune cells was lower in the high-risk group than in the low-risk group, and a portion of these immune cells were tumor-antagonistic. In addition, we found a relationship between these DRLs and the number of immune cells. According to the results of our research, the new DRLs signature has the potential to be utilized in the process of evaluating the tumor immune microenvironment in GC patients.

Tumor Mutation Burden (TMB) is considered as a key driving factor for the immunogenicity of neoantigens presented on major histocompatibility complex (MHC) on the surface of tumor cells, which affects patients’ response to immune checkpoint inhibitors (ICIs) [43]. Studies have shown that patients with high TMB tend to have good survival rates [44]. In this study, we found that prognostic characteristics were negatively correlated with TMB. Patients with low-risk scores had higher TMB, and patients with high TMB had better OS than those with low TMB. In contrast, survival analysis combining TMB and risk score demonstrated that patients with high TMB and a high-risk score had reduced prognostic value, and risk score based on DRLs was an independent factor influencing patients’ prognosis. Thus, risk scores based on disulfidptosis-related lncRNAs may screen populations that could benefit from immunotherapy.

Drugs such as 5-Fluorouracil, cisplatin, docetaxel, oxaliplatin and paclitaxel have been used in the clinical treatment of GC [45]. We used pRRophetic R package for the drug prediction. The IC50 represents the concentration of a therapeutic agent at which half of its inhibitory effect is observed. In our study, we observed that patients were more sensitive to anticancer drugs in the low-risk group. The results suggested that the DRLs risk signature may achieve good performance in predicting GC patients in response to drug therapy, while the specific molecular mechanisms needs further investigation. Taken together, the newly established DRLs signature is practicable for predicting clinical prognosis and has certain guiding implications in the treatment of immunotherapy and chemotherapy of GC. We also identified candidate small molecule compounds that can be characterized based on DRLs signature by the CMap database. Among them, lapatinib is a dual inhibitor targeting EGFR and HER-2, which demonstrated possibilities in preclinical studies of HER-2-positive patients with advanced gastric cancer [46]. Brivanib is a dual-target inhibitor of vascular endothelial growth factor receptor and fibroblast growth factor receptor. Recent studies have revealed that Brivanib can inhibit angiogenesis and target cGAS to enhance the anti-tumor immune response [47]. Our findings indicated that this signature has the potential to aid the discovery and identify therapeutic drug targets for the treatment of gastric cancer, which need further studies.

Inevitably, there are some limitations in this study. Firstly, the DRLs risk model was developed and validated using retrospective data from a public database. Therefore, prospective studies were essential to confirm and validate our findings and clinical efficacy of the risk model in the future. Secondly, although we firstly established the predictive risk signature based on disulfidptosis-related genes, some crucial clinical information was absence in the dataset, which limited the comprehensive understanding of the prognosis and therapeutic effects of GC. Thirdly, in this study, we divided the data into a training set and a test set. Although the statistical consistency of clinical indicators can be achieved by reducing the sample differences between the two groups, it would still bring some potential deviations for confounding factors and unmeasurable variables. Fourthly, due to the various sample sources, tumor heterogeneity and diverse detection methods, we were unable to detect and analyze all the relevant lncRNAs used for modeling, and thus lacked external cohorts, which need to be further explored in the future. Finally, to better understand the knowledge of disulfidptosis-related disorders, further in vivo and in vitro functional experiments are also necessary to explore the potential mechanisms of DRLs in GC, which will be our future direction.

## 5. Conclusions

In conclusion, our comprehensive study disclosed the molecular profile of DRLs and their clinical significance in GC. By constructing a novel disulfidptosis-related lncRNAs signature for GC patients, it accurately predicted the survival prognosis of GC, and better assessed the treatment efficacy of immunotherapy and chemotherapy. Therefore, the new DRLs model provided new insights for understanding the development and progression of GC and guiding individualized treatment strategies for GC patients. 

## Data Availability

The datasets presented in this study can be found in online repositories. The names of the repository/repositories and accession number(s) can be found in this article/Supplementary Material.

## Supplementary Materials

The following supporting information can be downloaded at https://www.mdpi.com/article/10.3390/biomedicines11123165/s1. Figure S1: The study wokflow; Figure S2: Relationship between disulfide death-related lncRNA and immunotherapy response; Figure S3: The correlation of SLC7A11 with risk models; Table S1: Clinicopathological characteristics of patients from TCGA-STAD cohort; Table S2: Disulfidptosis related gene; Table S3: Differential expression of lncRNAs between GC and normal tissues; Table S4: The correlation between disulfidptosis related gene and lncRNAs; Table S5: small molecular compounds for the treatment of gastric cancer from the CMap database.

## Abbreviations

AUC	Area under the curve
BP	Biological processes
CI	Confidence intervals
CC	Cellular components
DRLs	Disulfidptosis-related lncRNAs
GC	Gastric cancer
GSEA	Gene Set Enrichment Analysis
GO	Gene Ontology
lncRNAs	Long Non-coding RNAs
KEGG	Kyoto Encyclopedia of Genes and Genomes
K–M	Kaplan–Meier
LASSO	Least Absolute Shrinkage and Selection Operator
MF	Molecular functions
OS	Overall Survival
PFS	Progression Free Survival
ROC	Receiver operating characteristic
ssGSEA	Single-sample Gene Set Enrichment Analysis
TME	Tumor Microenvironment
TCGA	The cancer Genome Atlas
TMB	Tumor Mutation Burden
