# Supplementary material for: Comprehensive Analysis of Disulfidptosis-Related LncRNAs in Molecular Classification, Immune Microenvironment Characterization and Prognosis of Gastric Cancer
Source: Biomedicines. 2023 Nov 28;11(12):3165. doi: 10.3390/biomedicines11123165 (PMC10741100; doi:10.3390/biomedicines11123165)
Supplement: Supplementary file 1 [file biomedicines-11-03165-s001.zip › Supplementary Figure S1.pdf]

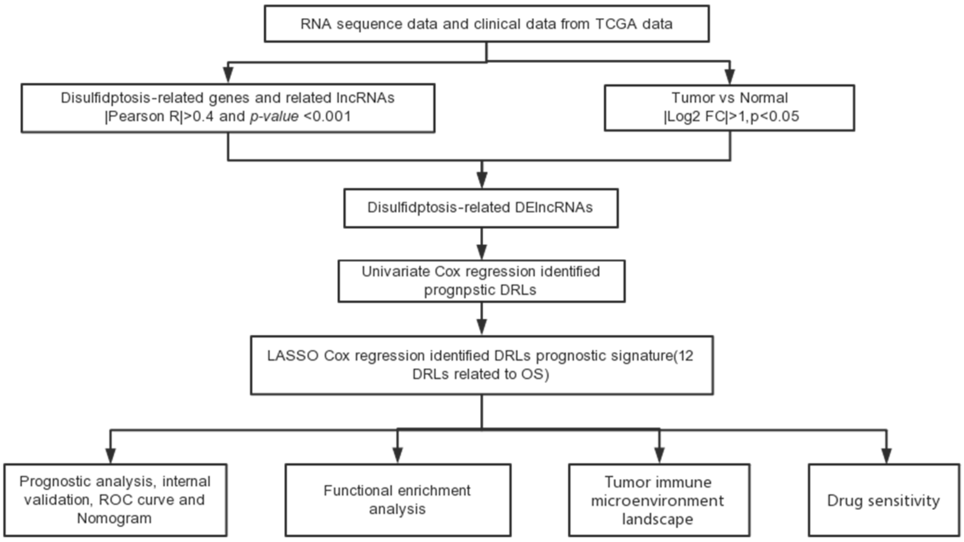

Supplementary Figure S1 The study workflow.

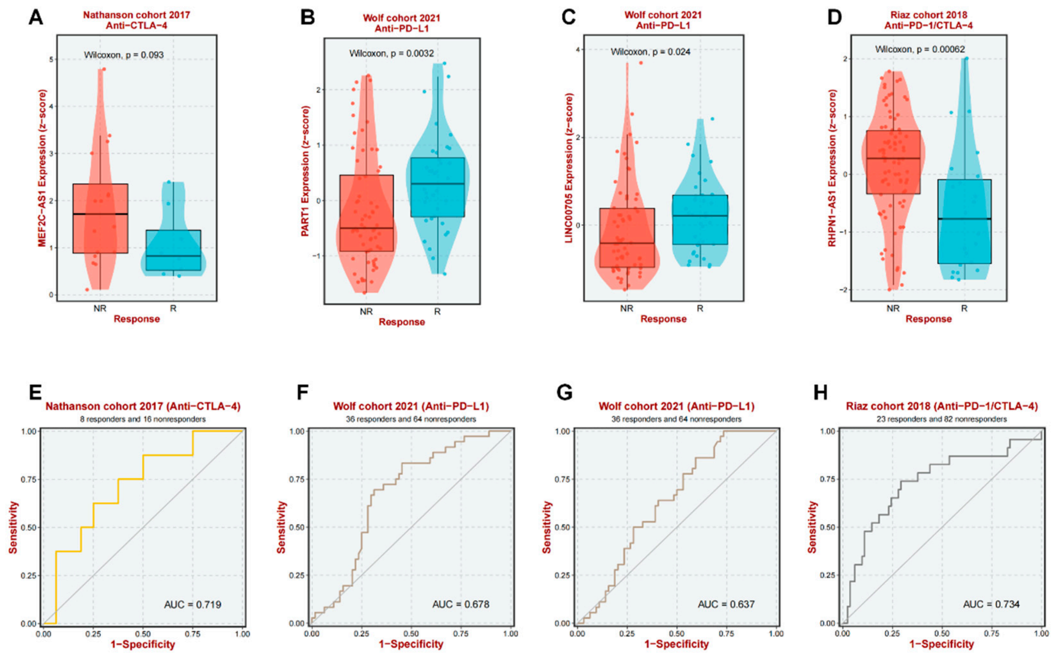

Supplementary Figure S2 Relationship between disulfide death-related lncRNA and immunotherapy response. **(A-D)** Expression of MEF2C-AS1, PART1, LINC00705 and RHPN1-AS1 in responders and non-responders based on immunotherapy cohort. **(E-H)** The ROC curve of MEF2C-AS1, PART1, LINC00705 and RHPN1-AS1 for patients in immunotherapy cohort.

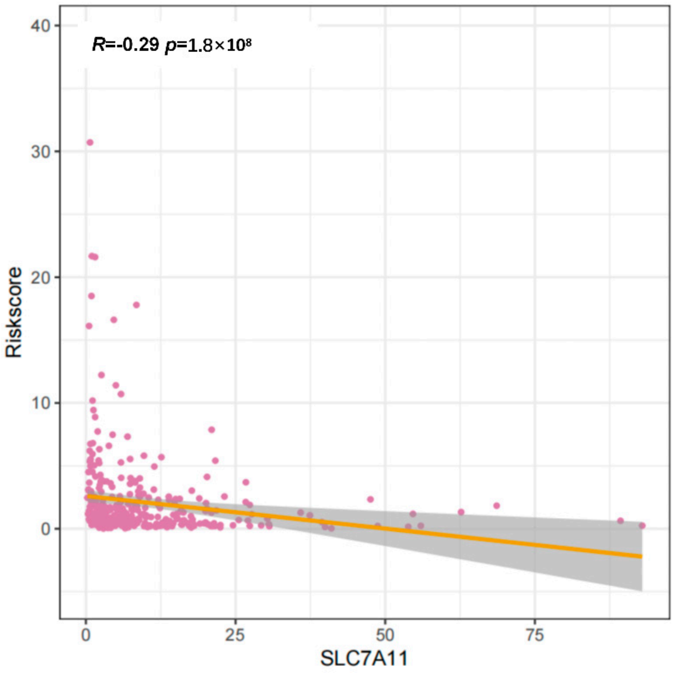

Supplementary Figure S3 The correlation of SLC7A11 with risk models.
