# Supplementary material for: Comprehensive Analysis of Disulfidptosis-Related LncRNAs in Molecular Classification, Immune Microenvironment Characterization and Prognosis of Gastric Cancer
Source: Biomedicines. 2023 Nov 28;11(12):3165. doi: 10.3390/biomedicines11123165 (PMC10741100; doi:10.3390/biomedicines11123165)
Supplement: Supplementary file 1 [file biomedicines-11-03165-s001.zip › Supplementary Table S1.pdf]

Supplementary Table S1. Clinicopathological characteristics of patients from TCGA-STAD cohort.

Covariates	Type	Total	Test	Train	*p*value
Age	<=65	183(44.96%)	95(46.8%)	88(43.14%)	0.49
	>65	221(54.3%)	106(52.22%)	115(56.37%)	
	unknow	3(0.74%)	2(0.99%)	1(0.49%)	
Gender	FEMALE	144(35.38%)	67(33%)	77(37.75%)	0.3701
	MALE	263(64.62%)	136(67%)	127(62.25%)	
Grade	G1	12(2.95%)	5(2.46%)	7(3.43%)	0.1782
	G2	144(35.38%)	64(31.53%)	80(39.22%)	
	G3	242(59.46%)	130(64.04%)	112(54.9%)	
	unknow	9(2.21%)	4(1.97%)	5(2.45%)	
Stage	Stage I	55(13.51%)	32(15.76%)	23(11.27%)	0.7129
	Stage II	122(29.98%)	63(31.03%)	59(28.92%)	
	Stage III	167(41.03%)	82(40.39%)	85(41.67%)	
	Stage IV	39(9.58%)	20(9.85%)	19(9.31%)	
	unknow	24(5.9%)	6(2.96%)	18(8.82%)	
